# Imaging in population science: cardiovascular magnetic resonance in 100,000 participants of UK Biobank - rationale, challenges and approaches

**DOI:** 10.1186/1532-429X-15-46

**Published:** 2013-05-28

**Authors:** Steffen E Petersen, Paul M Matthews, Fabian Bamberg, David A Bluemke, Jane M Francis, Matthias G Friedrich, Paul Leeson, Eike Nagel, Sven Plein, Frank E Rademakers, Alistair A Young, Steve Garratt, Tim Peakman, Jonathan Sellors, Rory Collins, Stefan Neubauer

**Affiliations:** 1 Centre Lead for Advanced Cardiovascular Imaging, William Harvey Research Institute, NIHR Cardiovascular Biomedical Research Unit at Barts, The London Chest Hospital, Bonner Road, London E2 9JX, UK; 2 Division of Brain Sciences, Department of Medicine, Imperial College, London, UK; 3 GlaxoSmithKline Research and Development, Ltd, Munich, Germany; 4 Department of Radiology, Ludwig-Maximilians University Munich, Munich, Germany; 5 Radiology and Imaging Sciences, NIH Clinical Center, Oxford, UK; 6 Division of Cardiovascular Medicine, Radcliffe Department of Medicine, University of Oxford, Oxford, UK; 7 Montreal Heart Institute, Université de Montréal and University of Calgary, Calgary, Canada; 8 King’s College London British Heart Foundation Centre of Excellence; National Institute of Health Research (NIHR) Biomedical Research Centre at Guy’s and St. Thomas’ (NHS)Foundation Trust; Wellcome Trust and Engineering and Physical Sciences Research Council (EPSRC) Medical Engineering Centre; Division of Imaging Sciences; The Rayne Institute, St. Thomas’ Hospital, London, UK; 9 Multidisciplinary Cardiovascular Research Centre & Leeds Institute of Genetics, Health and Therapeutics, University of Leeds, Leeds LS2 9JT, UK; 10 University Hospitals Leuven, Leuven, KU, Belgium; 11 Department of Anatomy with Radiology, University of Auckland, Auckland, UK; 12 UK Biobank, Spectrum Way, Adswood, Stockport, Cheshire SK3 0SA, UK

**Keywords:** Cardiovascular magnetic resonance, Prospective cohort study, Population-based study, Nested-case control study, Biobank

## Abstract

UK Biobank is a prospective cohort study with 500,000 participants aged 40 to 69. Recently an enhanced imaging study received funding. Cardiovascular magnetic resonance (CMR) will be part of a multi-organ, multi-modality imaging visit in 3–4 dedicated UK Biobank imaging centres that will acquire and store imaging data from 100,000 participants (subject to successful piloting). In each of UK Biobank’s dedicated bespoke imaging centres, it is proposed that 15–20 participants will undergo a 2 to 3 hour visit per day, seven days a week over a period of 5–6 years. The imaging modalities will include brain MRI at 3 Tesla, CMR and abdominal MRI at 1.5 Tesla, carotid ultrasound and DEXA scans using carefully selected protocols. We reviewed the rationale, challenges and proposed approaches for concise phenotyping using CMR on such a large scale. Here, we discuss the benefits of this imaging study and review existing and planned population based cardiovascular imaging in prospective cohort studies. We will evaluate the CMR protocol, feasibility, process optimisation and costs. Procedures for incidental findings, quality control and data processing and analysis are also presented. As is the case for all other data in the UK Biobank resource, this database of images and related information will be made available through UK Biobank’s Access Procedures to researchers (irrespective of their country of origin and whether they are academic or commercial) for health-related research that is in the public interest.

## Review

The challenges of understanding the determinants of common life-threatening and disabling diseases, such as myocardial infarction and stroke, are substantial. Such conditions are typically caused by many different risk factors that each have moderate effects and interact with each other in complex ways. Prospective cohort studies allow risk factors to be assessed before disease or its management affects participants, while diseases can also be assessed that are not readily investigated by retrospective studies. Prospective studies do, however, require involvement of large numbers of participants because only a relatively small proportion will develop a particular condition. When it is known which individuals have developed some particular condition then detailed analyses (e.g. of stored images) can be focused on them and on matched controls in so-called “nested” case–control studies.

This review describes the rationale, the challenges and the approaches of performing cardiovascular magnetic resonance imaging scans, as part of a multi-organ multi-modality imaging visit, on 100,000 of the 500,000 participants in the UK Biobank prospective cohort study.

### UK Biobank and CMR: current state and how did we get there?

UK Biobank (http://www.ukbiobank.ac.uk) has involved the collection of extensive baseline questionnaire data, physical measurements and biological samples from 500,000 men and women aged 40–69 at baseline between 2006 and 2010 in 22 centres across the UK. UK Biobank’s scientific protocol and operational procedures were reviewed and approved by the North West Research Ethics Committee (REC Reference Number: 06/MRE08/65). Participants’ health is now being followed long-term through linkage to health record systems and re-contact with participants. When reviewing the UK Biobank protocol in July 2006, the International Peer Review Panel recommended that more intensive phenotyping be conducted among large subsets of the cohort, including more detailed imaging assessments.

Since 2008, the UK Biobank Enhancement Working Group has invited imaging experts of different specialities to advise on their respective fields. From these experts, the UK Biobank’s Imaging Working Group emerged. The imaging experts were provided with the following brief: to build the strongest scientific case for using certain imaging modalities and protocols so that imaging can be achieved in 100,000 of the 500,000 UK Biobank subjects as part of a comprehensive enhancement visit, including imaging of at least the brain, heart, whole body, carotid arteries, bones and joints. The rationale for 100,000 participants is based on the requirement for 5,000 to 10,000 cases of some particular disease to allow reliable detection of odds ratios of about 1.5 to 1.3 for associations with various risk factors. For example: for the combination of myocardial infarction and death related to coronary artery disease, the expected numbers of incident disease occurring during follow-up of 100,000 imaged participants in UK Biobank would be 3,000, 6,000 and 10,000 in 2020 (5 year follow-up), 2025 (10 year follow-up) and 2030 (15 year follow-up), respectively.

In May 2009 UK Biobank submitted a proposal for various enhancements to the baseline visit that also included a proposal for conducting imaging assessments in dedicated regional centres. At that time, the funders deferred judgement on the imaging enhancement and recommended UK Biobank develop their imaging concept by wide-ranging consultation with the scientific imaging community. During the past few years, the Imaging Working Group has consulted extensively with imaging experts throughout the UK and internationally in a number of meetings and online discussions and established several international expert advisory groups (e.g. the CMR advisory group). The focus of the consultation process was to select and justify the most suitable imaging modalities and protocols. Additionally, the working group also discussed and developed plans of how best to store data, how to make data accessible for image analysis and how to ensure quality control.

Advisory groups for each imaging modality were tasked with developing protocols that would maximise the amount of relevant information obtained while fitting into per-participant time constraints that would allow imaging on a large scale. The Imaging Working Group has been responsible for developing the detailed research protocol and, in collaboration with UK Biobank, has been successful in being awarded the funding to start this imaging project of unprecedented scale. The project is planned in two stages: the first phase will take place over 12 to 18 months in one centre involving approximately 6,000 subjects to pilot feasibility and gather evidence related to the procedures of management of incidental findings. If successful, then the second phase will take place in 3–4 imaging centres to complete the imaging in 100,000 participants over a 5–6 year period.

### Choice of cardiovascular imaging modality

The brief described above was presented to the imaging experts to advise on which modality or modalities would be best to achieve large-scale cardiovascular phenotyping as part of a multi-organ imaging assessment. This led to the further refinement of a framework which focuses on the following: 1) the safety and comfort of participants was considered essential; 2) the combined visit should not exceed 3 hours; 3) a recruitment rate of at least 20% would need to be achieved with a low rate of non-compliance or drop-out during the visit and 4) for cost-effectiveness and quality control reasons, imaging would need to take place in dedicated UK Biobank regional imaging centres using uniform equipment rather than a hospital setting. From this framework, the following list of requirements regarding the cardiovascular imaging modality was developed: the test would need to be non-invasive, not use radiation, should not require the administration of medications or contrast agents, last at most 20 minutes, parameters acquired should be reproducible, and include the most relevant quantifiable cardiovascular imaging parameters.

As cardiac CT uses radiation and would require contrast administration and often beta blockade for CT coronary angiography, this modality was not considered further for this population-based setting. Extensive discussions were held around whether echocardiography, CMR or both should be recommended. Following expert presentations to the UK Biobank International Scientific Advisory Board meeting in 2011, the decision was made to support CMR alone as part of the imaging assessment. This decision was based on the better reproducibility of CMR (which is also beneficial for the power of the study), the likelihood of better quality control, and the number and importance of the relevant cardiovascular parameters that can be derived with a 20-minute protocol. The main arguments for echocardiography were based around lower cost and imaging of valve structure and function. For this large-scale high-throughput model, however, the marginal cost for CMR (despite the high up front capital cost) compared to echocardiography was estimated to be approximately £50/subject only and valve pathology, a strength of echocardiography, was not considered as relevant for the UK Biobank population as the CMR derived parameters.

### Existing prospective cohort studies using CMR

Previous cohort studies with CMR have involved only a few thousand participants (Table 1); studies of this size are mainly designed to estimate the prevalence of disease and of risk factors since too few of the participants subsequently develop any particular condition to study the associations of risk factors with subsequent disease. The largest published population-based investigation that involves CMR is the Multi-Ethnic Study of Atherosclerosis (MESA). In this multicentre cohort study, 5004 asymptomatic individuals from four ethnic groups in the USA have had traditional CMR measures of left and right ventricular (LV/RV) mass, volume and function and aortic function. Subgroups have also had additional CMR modalities, including: tagging to assess regional wall motion, contractility and relaxation (about 1200 participants); contrast-enhanced perfusion CMR (n = 222); and coronary artery MR (n = 180). Study participants were enrolled from 2000–2002, and most of the analyses derived from these CMR data have been cross-sectional. For example, MESA has examined the gender and ethnic distributions of LV and RV volume, mass and ejection fraction [1,2], and associations of traditional and novel cardiovascular risk factors (such as inflammatory markers) with global and regional systolic and diastolic cardiac function [3-11]. Novel associations have been identified between cardiovascular risk factors and CMR parameters [12,13]. Since multiple imaging modalities were included, MESA allows the comparison of CMR findings to coronary artery calcium (CT scanning) and carotid intimal media thickness [14]. MESA has shown high reproducibility of tagging [15], and associations between vascular function, cardiac function and myocardial blood flow [16-19]. The 10-year follow-up of MESA has recently been completed, enrolling approximately 3013 of the original 5004 CMR participants. Of these, 1839 subjects had gadolinium enhanced CMR studies.

**Table 1:** Previously developed prospective population studies with CMR imaging (including at least 1000 participants)

	**Age of cohort (y)**	**MRI brain**	**CMR**	**MRI body**
Jackson Heart Study	35-84		2,000	
SHIP	20-79		4,000	4,000
MESA	45-84		5,000	
Framingham Heart Study	38-88	2,500	1,800	
Dallas Heart Study	18-65	3,000	3,000	3,000
AGES Reykjavik	>70	5,000	1,000	

CMR: Cardiovascular Magnetic Resonance.

The Dallas Heart Study involves CMR at 1.5 Tesla in 3000 people aged 18 to 65 years from a multi-ethnic population in which global LV function, volumes, mass and gradient echo cine sequences (which are now outdated) have been measured in over 90% of subjects [20-22]. The East German population-based Study of Health in Pomerania (SHIP) is currently acquiring whole-body MRI at 1.5 T in approximately 4000 healthy participants aged 20 to 79 years. The CMR aspect involves the assessment of global and regional cardiac function, with optional late gadolinium enhancement to identify myocardial scar or fibrosis [23,24]. The Jackson Heart Study is currently acquiring CMR scans in about 2000 African-Americans aged 35–84 years. The measures include the assessment of global and regional cardiac function, late gadolinium enhancement to identify myocardial scar or fibrosis and measures of aortic structure and function.

### Enhanced phenotyping with multi-modality, multi-organ imaging in UK Biobank

Following the consultation process described above, the agreed imaging enhancement visit will include MRI of the brain, heart, aorta, abdomen and carotid ultrasound and Dual-energy X-ray absorptiometry (DEXA). By contrast with previous studies, the integration of large-scale data in UK Biobank from imaging one part of the body with imaging data from other parts of the body, as well as with the detailed non-imaging data (environmental factors, genotypes etc.) that have already been collected, will support a unique approach to the investigation of the biological mechanisms of disease. For example, cognitive decline (such as dementia) may be related to risk factors derived not only from the brain, but also from the heart (e.g. variations in structure and function [25-27], arteries, carotid plaques [28-30], body fat [31], bones and joints [32]). Identifying these intertwining risk factors will be made possible with the use of the UK Biobank multi-modality imaging resource.

### CMR approach in UK Biobank

**Figure 1:**
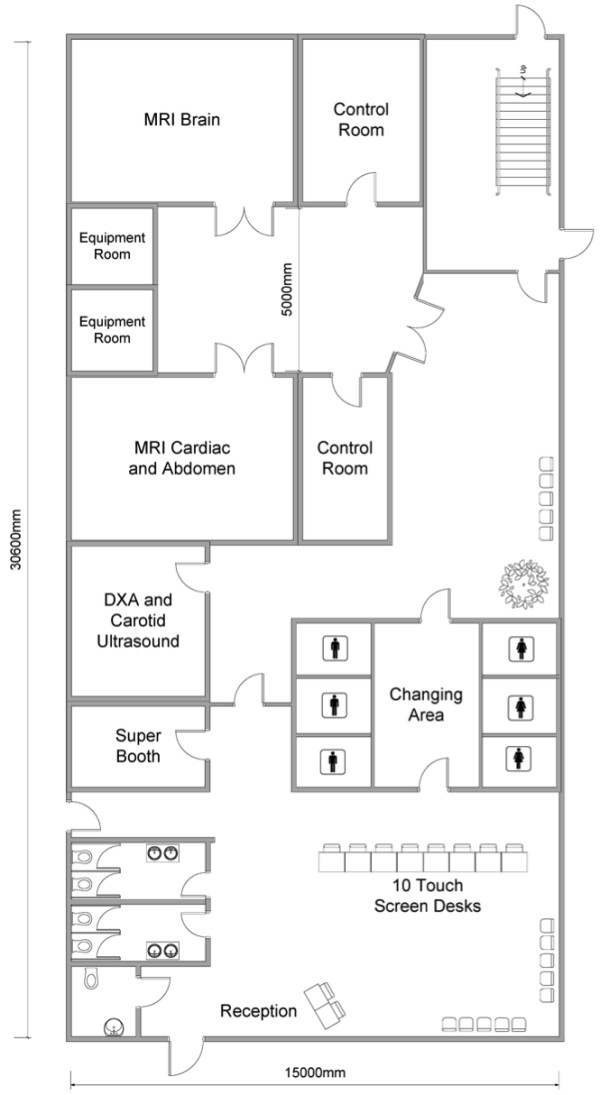
Dedicated UK Biobank imaging centre layout.

The CMR protocol was developed bearing in mind the framework and requirements. Each participant will undergo a 20-minute CMR protocol, as part of a 30-minute combined CMR and abdominal MRI protocol, using a 1.5 Tesla scanner that will include steady state free precession cine imaging in long axis, ventricular and atrial short axes and axial planes through the aorta, myocardial tagging and aortic flow sequences. This protocol will allow the determination of the minimum dataset in Table 2. Each participant’s visit would also include a 30-minutes brain MRI at 3 Tesla, a DEXA scan (10–15 minutes), and 3D carotid ultrasound (10–15 minutes) in addition to preparation (including consenting) and collection of non-imaging data and biological samples (e.g. partial repeat of the baseline assessment visit with supplementary cognitive function tests). The notional layout of a dedicated UK Biobank imaging centre is displayed in Figure 1.

**Table 2:** Minimum dataset of parameters that will be able to be derived from images acquired in UK Biobank CMR protocol

**Cardiovascular structure**	**Quantifiable parameters**
**Left ventricle**	Myocardial mass (g), ejection fraction (%), end-diastolic volume (ml), end-systolic volume (ml), stroke volume (ml) and the corresponding values indexed to body surface area, height or weight. Time to peak contraction and filling rates (both in s) and peak contraction and filling rates (both in ml/s). *American Heart Association (AHA) myocardial segments:* end-diastolic thickness (mm), end-systolic thickness (mm), thickening (mm), thickening (%). *Strain*: strain (%) and strain-rates (1/s) in three directions (radial [E_RR_], circumferential [E_CC_] and longitudinal [E_LL_]) and in systole and diastole and corresponding changes in angle caused by shear (E_RC_, E_RL_ and E_CL_).
**Right ventricle**	Myocardial mass (g), ejection fraction (%), end-diastolic volume (ml), end-systolic volume (ml), stroke volume (ml) and the corresponding values indexed to body surface area, height or weight.
**Left atrium**	End-diastolic volume (ml), end-systolic volume (ml), stroke volume (ml), ejection fraction (ml) and left atrial diameters as typically measured by 2D-echocardiography approaches.
**Right atrium**	End-diastolic volume (ml), end-systolic volume (ml), stroke volume (ml), ejection fraction (ml) and right atrial diameters as typically measured by 2D-echocardiography approaches.
**Aorta**	Distensibility (1/mmHg) in ascending aorta, proximal descending and distal descending aorta; diastolic aortic dimensions (cm^2^): ascending aorta, proximal descending and distal descending aorta. Proximal, distal and total pulse wave velocity (m/s).

UK Biobank’s Cardiovascular MRI Advisory Group will continue to advise on data acquisition, radiographer training and quality control, as well as providing guidance on subsequent applications for research uses. The CMR protocol is subject to modifications before, during and immediately after the pilot phase, but it is not likely to be changed subsequently during the main phase of the study. Examples of opportunities for modification include automated plane finding algorithms or full 3D volumetric acquisition of the heart [33]. CMR feature tracking, which is similar to echocardiographic speckle tracking, tracks regional myocardial features throughout the cardiac cycle. This technique allows myocardial strain measurements to be derived without the need for additional imaging sequences (such as tagging) because features are tracked from clinically standard steady-state free precession (SSFP) sequences [34]. If CMR feature tracking is sufficiently validated and robust when the UK Biobank imaging assessments take place then it may be possible to remove the tagging sequences from the protocol and use that time for other sequences (such as mitral and aortic flow sequences or non-contrast T1 mapping techniques, which have recently been shown to be highly reproducible and differentiate between healthy controls and patients with cardiomyopathies) [35-38].

There was agreement among the experts consulted that this non-contrast CMR protocol would not benefit in quality from using a magnetic field strength above 1.5 T. Higher field strengths would render it more expensive and less feasible, largely due to prolonged image optimisation and shimming requirements. Contrast agent sequences would provide important extra information (e.g. myocardial blood flow, myocardial fibrosis/scar and LV thrombus), but experts agreed it was not feasible to include MR-contrast in such a large population-based study. In particular, there was a risk (albeit small) of serious adverse events: among 100,000 participants, at least one death and up to 1000 other serious adverse reactions (depending on the definition) might be expected if contrast agents were administered. Other reasons for not using contrast included complexity of scan acquisition, associated technologist training needs and potential effect on brain MRI scans.

### Feasibility, process optimisation and costs

**Figure 2:**
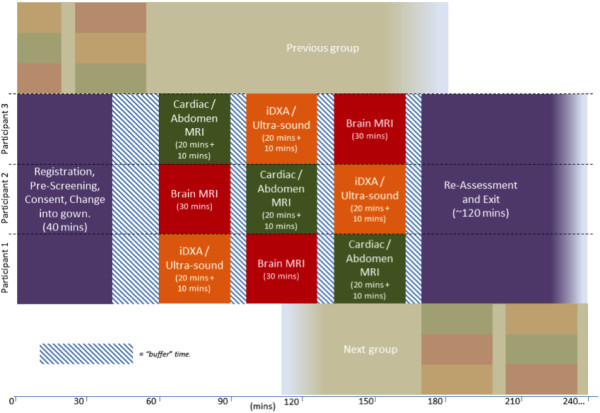
Representation of participant flow with complex buffered process for groups of 3 participants.

The imaging protocols are all based on established technologies and each has been pre-piloted to confirm its feasibility within the time constraints. Modelling indicates that 100,000 participants could be imaged within 6 years in 3–4 dedicated imaging centres. A 12 – 18 month pilot phase will first be conducted in one of the imaging centres to establish optimal participant response rates, imaging assessment throughput, and data storage. Participants’ understanding of the consent process and procedure for feedback of incidental findings will also be evaluated in the pilot. This would be followed by a main phase of 5 years with 3–4 concurrently operating dedicated UK Biobank imaging centres (Table 3). The target is to image 21 participants per centre per day using a complex buffered approach (Figure 2) and imaging centres operating for 14 hours per day, 7 days per week. The numbers of staff with different levels of expertise required to achieve such a throughput in a shift system is summarised in Table 4.

**Table 3:** Estimated number of participants to be imaged and operational duration in each of the three imaging centres for the UK Biobank imaging enhancement

	**Total number of participants recruited in baseline**	**Approximate anticipated attendance rate**	**Recruited participants**			**Centre operational duration (years)**			**Total duration of Project (years)**
			Pilot	Main	Total	Pilot	Main	Total	
North	150,601	25.0%	8,520^1^	29,130	37,650	1.5	4.1	5.6	-
South	155,545		-	38,886	38,886	-	5.5	5.5	-
N. East + Scotland	94,233		-	23,558	23,558	-	3.3	3.3	-
	**400,379**				**100,095**			**14.4**^**2**^	**7.0**

^1^ Assumes the pilot processes an average of 16 participants a day, and operates 355 days of the year for 18 months.

^2^ Assumes we can process an average of 20 participants a day for the main study, operating 355 days of the year.

**Table 4:** Estimated staffing mix needed for a single imaging centre

**Staff role and grade**	**Responsibility**	**Number**
Manager (NHS Band 9)	Day-to-day management, training, support, efficient operation of the centre, participant assessment	3
Radiographer (NHS Band 8)	Brain/cardiac/abdominal MR imaging	10
Technician (NHS Band 6)	DXA and 3D carotid ultrasound	3
Receptionist	Participant welcome, registration and visit coordination	5

Staff “number” refers a pool of staff working shifts to cover 14 hour day and seven day week operation.

Subject to successful piloting in 2013/14 at a cost of about £10 M (which includes the full capital costs for setting up the first dedicated imaging centre, the main phase of imaging assessments would be conducted during 2014–2018 at a further cost of about £27 M, yielding 100,000 participants with imaging enhancements at a total cost of £37 M. This cost works out at less than £400 per participant for all of the different imaging data and compares favourably with the costs of imaging in previous epidemiological studies. In particular, the CMR component is less than £100, which compares favourably to the much higher costs of clinical CMR studies.

These relatively low costs can only be achieved through the specific UK Biobank imaging centre set-up. These imaging centres (Figure 1) will be rented or purpose-built on the basis of feasibility and cost of scanning 100,000 participants. Factors influencing the choice of location include reasonable distance to participants’ homes, transport connections, power supply, building or renting costs.

### Quality control of CMR images

One of the most important requirements for the success of a massive-scale imaging study such as UK Biobank imaging enhancements is a vigorous, accountable and sustained quality control process. The radiographers will have extensive training on the imaging platform provided by the vendor. Prior to the start of the pilot phase, radiographers will have further training in a high-volume CMR unit, including observation and hands-on CMR acquisition. Subsequently, these radiographers will be trained in the imaging centre set-up for UK Biobank before starting to work in the pilot phase or in the main phase of imaging enhancements. The radiographers will perform quality checks of the images that are obtained (up to about 10% of images during the initial pilot phase, but likely to decrease to 2-5% with greater experience). Some time from a dedicated MR physicist and CMR consultant will be supported for review of subsets of the images. The MR physicist will arrange regular MR phantom measurements as part of quality control procedures. The CMR consultant will visit the imaging centres regularly to ensure acquisition standards are met and to provide additional training on site as necessary. Radiographers for the additional dedicated imaging centres during the main phase will be trained in the pilot phase imaging centre.

### Data processing and access procedures

It is intended to apply available automated analysis tools to the imaging data at the time of collection and to conduct detailed non-automated analyses only subsequently in a targeted way. So, for example, when sufficient numbers of people have developed some particular condition, specific analyses would be conducted of the relevant participant images and matched controls. Such nested case–control comparisons would provide most of the statistical information about the association of particular image-related factors with the particular health outcome, while being much more cost-effective than conducting non-specific image analyses in all participants at the time of image collection. In addition, it is likely that CMR image analysis during the next 5–10 years will become more automated and cheaper, and that recommendations to standardise image analysis will be published.

Researchers will be able to apply for access to image data for specific scientific questions in accordance with UK Biobank’s Access Procedures (http://www.ukbiobank.ac.uk). There is a two stage application process involving a review by UK Biobank of the preliminary application and the provision of a suitable quote (which is intended to assist researchers with the writing of a grant to cover such costs). Quotes will include costs – charged on a cost recovery basis by UK Biobank - for gaining access to images for analysis, for requesting image data that have already been analysed and for requesting other variables from the tests and questionnaires in UK Biobank. If researchers are granted access to UK Biobank data or images, they are obliged to return the results of their analysis to UK Biobank’s database following the completion of their project. In turn, this will result in the creation of a more detailed resource of imaging information within UK Biobank, which will be able to be accessed by other researchers.

UK Biobank CMR imaging provides a platform for new opportunities. Recent advances in machine learning algorithms are providing encouraging results that indicate that entirely automated analysis of cardiac function may be available even within the initial phases of the UK Biobank imaging enhancements [39]. The Cardiac Atlas Project combined images and results from MESA and the Jackson Heart Study to enable meta-analyses of probabilistic cardiac shape and function patterns [40,41]. This could be extended to the larger number of participants in UK Biobank. By mapping the regional function of the heart using consistent coordinates, mathematical models of heart shape and motion can be used to form an atlas of regional shape and motion.

### Incidental findings

A significant challenge for large-scale, and in particular multi-organ, imaging studies in a mostly healthy population living in the community is how to deal with incidental findings [42]. It is beyond the scope of this review to provide an in-depth analysis of the legal and ethical issues related to dealing with incidental or unexpected findings. However, a recent report by representatives of research imaging centres, which included professional societies, regulatory bodies, funding organisations and patient organisations in the UK, summarises the position succinctly “the challenge is to strike the correct balance between answering the research question and the welfare of the participant… Research to clarify these issues is vital for developing evidence-based policies for the management and feedback of incidental findings” [43].

The approach to incidental findings proposed by UK Biobank has been very carefully considered and took into account evidence from literature reviews, experiences from other population-based imaging research projects, discussions with the Royal College of Radiologists and the Society and College of Radiographers and legal advice. In addition, input was obtained from UK Biobank’s independent Ethics & Governance Council. UK Biobank’s provisional approach will be to provide limited feedback for incidental findings considered to be potentially ‘serious’ (defined in this context as likely to threaten life span, quality of life or major body functions) that are observed during the data acquisition or quality control stage. The feedback loop provides for review by expert radiologists and then, if appropriate, feedback will be provided to the participant and his/her general practitioner. This approach to imaging incidental findings is consistent with – but more detailed than - the standard operating procedure on incidental findings used in the baseline UK Biobank assessment visit. Further, since the imaging procedures increase the potential likelihood for serious incidental findings to be observed, detailed standard operating procedures will be developed and then tested during the pilot study. Participants will be given detailed and explicit information about the imaging assessment (including the approach to feedback of incidental findings) before giving consent. Their understanding of UK Biobank’s approach and its impact on them and the wider health community (including induced healthcare costs) will also be assessed carefully during the pilot phase.

## Conclusions

This review describes the rationale, challenges and proposed approaches for the imaging enhancements of the UK Biobank population based cohort study involving multiple imaging modalities including brain, body composition, bone, joints and, as focussed on here, heart and large blood vessels. This large-scale study is intended to provide sufficient statistical power for reliable assessment of associations between these imaging phenotype measures and a wide range of incident diseases. As with the successful recruitment and baseline assessment phase of UK Biobank, the application of industrial processes to optimise and manage these imaging assessments will allow them to be conducted cost-efficiently. The pilot phase during 2013/14 is intended to develop streamlined procedures and confirm feasibility and costs. The main phase is intended to take place during 2014–2018.

As is the case for the data within the UK Biobank resource, all imaging data will be available to bona fide researchers conducting health-related research that is in the public interest.

## Abbreviations

AHA: American Heart Association; CMR: Cardiovascular magnetic resonance; MRI: Magnetic resonance imaging; NHS: National Health Service; UK: United Kingdom.

## Competing interests

SP receives grant support from Philips Healthcare. PMM is a part-time employee of GlaxoSmithKline Research and Development, Ltd.

## Authors’ contributions

All authors 1) have made substantial contributions to conception and design 2) have been involved in drafting the manuscript or revising it critically for important intellectual content; and 3) have given final approval of the version to be published.

## Authors’ information

Steffen E. Petersen, MD DPhil FESC FACC Reader in Advanced Cardiovascular Imaging, Honorary Consultant Cardiologist, Centre Lead for Advanced Cardiovascular Imaging, William Harvey Research Institute, NIHR Cardiovascular Biomedical Research Unit at Barts, The London Chest Hospital, Bonner Road, thor London, E2 9JX, UK.
